# Patient-reported experience and quality of care for people with schizophrenia

**DOI:** 10.1186/s12888-018-1998-y

**Published:** 2019-01-09

**Authors:** L. Aimola, J. Gordon-Brown, A. Etherington, K. Zalewska, S. Cooper, M. J. Crawford

**Affiliations:** 1 The Centre for Psychiatry, Imperial College London, Hammersmith Campus, Commonwealth Building 7th Floor, Du Cane Road, London, W12 0NN UK; 2 The Royal College of Psychiatrists’ College Centre for Quality Improvement, London, E1 8BB UK

**Keywords:** Psychosis, Patient satisfaction, Patient outcome measures, Quality improvement

## Abstract

**Background:**

Evidence is mounting that patient-reported experience can provide a valuable indicator of the quality of healthcare services. However, little is known about the relationship between the experiences of people with severe mental illness and the quality of care they receive. We conducted a study to examine the relationship between patient-reported experience and the quality of care provided to people with schizophrenia.

**Methods:**

We calculated a composite global rating of quality of care for people with schizophrenia using data from an audit of 64 mental health providers. We then examined associations between these ratings and mean patient satisfaction and patient-rated outcome using data from a survey of 5608 schizophrenic patients treated in these services.

**Results:**

Global rating of quality of care was positively correlated with patient-rated outcome (*r* = 0.33; *p* = 0.01) but not with patient satisfaction (*r* = 0.21, *p* = 0.10). Patient-rated outcome was also positively correlated with patient involvement (*r* = 0.26, *p* = 0.04) and the quality of prescribing practice (*r* = 0.31, *p* = 0.02). High patient satisfaction scores were significantly associated with the extent of use of care plans within each organisation (*r* = 0.27, *p* = 0.03).

**Conclusions:**

Among people with schizophrenia, patient-rated outcome provides a better guide to the quality of care than patient-rated satisfaction. Greater use of patient-reported outcome measures should be made when assessing the quality of care provided to people with psychosis.

## Background

Improving the quality of care that patients receive is a key objective for healthcare providers across the world. As well as safety and clinical effectiveness, patients experience of care is a key component of most definitions of quality of healthcare [1].

In recent years evidence has begun to emerge that patient-reported experience can offer a reliable indicator of the quality of care that services provide [2–4]. A systematic review summarising evidence from 55 studies, showed consistent positive associations between patient-reported experience and clinical effectiveness [2]. However, most studies included in this systematic review focused on patients using primary care and general hospital services. Only two studies included data from patients using mental health services and one study specifically excluded patients with a psychiatric diagnosis. It has been argued that assessment of patient experience of mental health services is challenging because people may not have sufficient information about what such services are able to provide [5], and because severe mental illness may impair a person’s insight [6].

Small scale studies have examined the relationship between patient-reported experience and other measures of quality of care. For instance, Shipley and colleagues [7] investigated the relationship between the quality of a community mental health service and patient-rated satisfaction with the care they received. Based on data from 113 randomly selected patients referred to this service over a two-month period, the authors found that patient-reported experience of care provided a more accurate indicator of quality of care than standard quality indicators such as waiting times. Although the results of this study support the use of patient experience as a measure of service quality in mental health settings, the generalisability of the results of study are limited by the small sample size and single site.

The second round of the National Audit of Schizophrenia conducted between 2013 and 2014 assessed the quality of care provided to patients with schizophrenia across all the Mental Health Trusts in England and Health Boards in Wales. As the audit provided data on a wide range of outcomes of care for patients with schizophrenia around the county, we set out to use data from this audit to examine whether patient-reported experience provides an indication of the services’ overall quality of care in a nationwide cohort of patients with severe mental health illness.

## Methods

The National Audit of Schizophrenia provided an in-depth examination of service performance against 16 standards of care derived from national guidelines for the treatment of people with schizophrenia in England [8]. These standards concern the quality of physical health monitoring, prescribing practice, use of evidence-based psychological treatments and patient involvement in care planning. Our study comprised a secondary analysis of cross-section data from an audit of clinical records of the quality of care provided to people with psychosis and the results of a patient survey that was conducted in parallel with the audit.

### Data collection

A total of 64 NHS Mental Health Trusts in England and Health Boards in Wales (referred to collectively as ‘Trusts’ in the remainder of this report) participated in the audit. Data were collected via an audit of practice tool and a contemporaneous patient survey. The audit of practice tool was developed specifically to measure standards of care and the relevant data were obtained from the patients’ case records (see Appendix for a list of the standards). The tool was completed by consultants, junior doctors and other allied health professionals within the services. The patient survey was developed to assess patient-reported experience of care. An expert patient reference group helped refine draft versions of both the audit of practice tool and the patient survey prior to the start of the audit.

Trusts were asked to complete the audit of practice tool for a random sample of 100 patients. In addition, Trusts were required to distribute the patient survey to 200 randomly selected patients including the 100 patients whose notes were examined using the audit of practice tool. The data collection was conducted between August 2013 and November 2013.

### Patient sample

Patients were eligible for inclusion in the audit if they were aged above 18, with a current ICD-10 diagnosis of schizophrenia or schizoaffective disorders. To be eligible for the audit patients had to have received treatment from community mental healthcare setting within the Trust for at least 1 year.

### A composite measure of global trust performance

For each Trust, we calculated the proportion of patients for whom each individual standard was met and the mean percentage of compliance for each standard relative to the total national sample. We then calculated a global trust rating score based on the mean percentage of compliance of each Trust across all standards assessed with the audit of practice tool. We also calculated five sub-scores from standards relating to:

1. The quality of physical health monitoring the patient received (e.g. assessments and interventions delivered).
2. The quality of patient involvement (e.g. whether there was shared decision making process in the selection of medication).
3. Compliance with recommended prescribing practice (e.g. extent of polypharmacy and prescribing above BNF recommended maxima).
4. Access and uptake of evidence-based psychological treatments.
5. Provision of a care plan jointly developed with the patients to meet their needs.

### Patient-reported experience measures

The patient-reported experience measures we used included a measure of patient satisfaction with care and a patient-rated outcome measure. The question on patient satisfaction with care asked: “Taking everything into consideration, are you pleased with the care you have received from the service so far?” Responders had to indicate their answer by using a four-points Likert scale from 1 (not satisfied at all) to 4 (very satisfied). The question on patient-rated outcome asked: “To what extent have services helped you to achieve good mental health in the last year?” and patients responded using a four-point Likert scale ranging from 1 (made me worse) to 4 (helped a lot). We calculated the mean trust scores for these two questions and converted them into a 100-point scale.

### Statistical analysis

We carried out Pearson product-moment correlations to investigate associations between patient satisfaction and patient-rated outcome scores with the global trust performance and subgroups of care scores [9].

## Ethics approval and consent to participate

The National Audit of Schizophrenia (NAS) was commissioned by the Healthcare Quality Improvement Partnership (HQIP) as part of the National Clinical Audit Programme (NCA). As part of NCA, all Mental Health Trusts in England and Wales were expected to take part in NAS. Consent was not required from the patients to publish the audit data in the audit’s main report or for research purposes and the data were collected by the audit team in such a manner that the patients could not be directly identified.

## Results

The audit team received 5733 returns from the audit of practice, of which 5608 were used in the analysis after data cleaning with a mean of 88 (SD = 17) returns per Trust. In total, 3379 patients submitted a survey form with a mean of 53 (SD = 19) per Trust and a response rate of 26.4%. Table 1 shows a breakdown of the demographic characteristics of the patients included in the audit of practice according to whether they did or did not respond to the survey. The demographic data on the responders and non-responders to the patient survey refer to the only two Trusts that were able to collect this information. The responders and not-responders group did not differ with regard to gender, ethnicity, and time since diagnosis.

**Table 1:** Demographic characteristics of the patients included in the audit and of those who responded and did not respond to the survey

Demographic characteristics	Patients included in the audit (*n* = 5608)	Responders to the survey (*n* = 61)^a^	Non responders (*n* = 240)^a^	Difference in proportions (95% CI)	*p* value
Age mean (Standard Deviation)	46 (13)	51 (11)	49 (12)	−2.0 (−5.3 to 1.4)	0.249
Gender n (%)					
Male	3655 (65.2%)	45 (73.8%)	153 (63.8%)	10.0	0.174
Female	1949 (34.8%)	16 (26.2%)	87 (36.3%)	(−3.5 to 21.3)	
Not stated	4 (< 0.1%)	0 (0.0%)	0 (0.0%)		
Ethnicity n (%)					
White	4400 (78.5%)	48 (78.7%)	159 (66.3%)	12.4 (−0.7 to 22.9)	0.500
Asian or Asian British	446 (8.0%)	3 (4.9%)	21 (8.8%)	−3.8 (− 9.2 to 5.2)	
Black or Black British	454 (8.1%)	8 (13.1%)	41 (17.1%)	−4.0 (−12.2 to7.5)	
Chinese or other	108 (1.9%)	1 (1.6%)	6 (2.5%)	−0.9 (−6.4 to 4.0)	
Mixed	116 (2.1%)	0 (0.0%)	5 (2.1%)	−2.1 (−4.8 to 4.0)	
Not stated	84 (1.5%)	1 (1.6%)	8 (3.3%)	−1.69 (−5.6 to 5.1)	
Time since diagnosis					
Between 1 and 2 years	226 (4.0%)	0 (0.0%)	4 (1.7%)	−1.7 (−4.4 to 4.2)	0.183
From 2 to 4 years	495 (8.8%)	3 (4.9%)	22 (9.2%)	−4.3 (−9.6 to 4.9)	
From 4 to 10 years	1353 (24.1%)	12 (19.7%)	67 (27.9%)	−8.2 (4.5 to −18.3)	
More than 10 years	3534 (63.0%)	46 (75.4%)	147 (61.3%)	14.1 (0.7 to 25.2)	
Care team n (%)					
Assertive Outreach	689 (12.2%)	3 (4.9%)	17 (7.1%)	−2.2 (−6.8 to 7.3)	0.085
Community Team	4035 (72.0%)	57 (93.4%)	194 (80.8%)	12.6 (2.4 to 19.4)	
Crisis Resolution	13 (0.2%)	0 (0.0%)	0 (0.0%)	–	
Early Intervention	239 (4.3%)	0 (0.0%)	4 (1.7%)	−1.7 (−4.3 to 4.2)	
Other	632 (11.3%)	1 (1.6%)	25 (10.4%)	−8.8 (−13.5 to 1.0)	

^a^ Data in these two columns refer only to the two trusts which collected information on responders and non-responders

### Association between patient-reported experience measures and overall trust rating

**Fig. 1:**
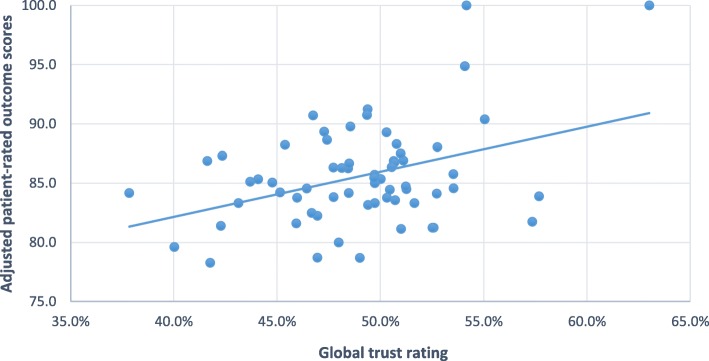
Scatterplot showing a positive linear correlation between global trust rating scores and patient-rated outcome scores among

There was a positive significant correlation between the patient satisfaction and the patient-rated outcome measure (rs = 0.582, *p* < 0.01). In Table 2, we present (i) correlations between patient-rated outcome and quality of care data from the audit, and (ii) correlations between patient-rated satisfaction and quality of care data from the audit. While patient-rated outcome scores were significantly associated with the global trust rating, patient satisfaction scores were not. Figure 1 shows the scatterplot of the relationship between global trust rating and patient-rated outcome about their mental health.

**Table 2:** Correlations between the global trust rating and its subgroups with patient satisfaction and patient experience

Composite measure	Correlation coefficient (*p* value)	
	Patient satisfaction	Patient-rated outcome
Global trust rating	0.209 (0.098)	0.329** (0.008)
Physical health monitoring	0.169 (0.182)	0.199 (0.114)
Patient involvement	0.149 (0.241)	0.263* (0.036)
Pharmacotherapy	0.106 (0.404)	0.310* (0.013)
Psychotherapy	−0.024 (0.850)	−0.002 (0.990)
Care planning	0.274* (0.029)	0.026 (0.838)

**p < 0.01

*p < 0.05

The results relating to the five subgroups of patient care we examined are also presented in Table 2. Only the provision of a care plan was significantly correlated with high patient satisfaction scores. In contrast, patient-rated outcome scores were positively correlated with patient involvement and with the services’ compliance with recommended prescribing practice. No significant associations were observed between the patient-reported experience measures and the other subgroups of patient care.

## Discussion

Secondary analysis of data from this national audit of care for people with schizophrenia found that patient-rated outcome was positively associated with an independent rating of the global quality of care provided by these services. In contrast, we did not find an association between global quality of care and the patients’ satisfaction with the service they received. High scores on patient-rated outcome were also correlated with high patient involvement in the decisions about their care and with high compliance of the services with the recommended prescribing practice. In contrast, high levels of patient satisfaction were associated only with the provision of a care plan tailored to the patients’ needs. Nonetheless the strength of these associations are not strong and caution needs to be exercised in interpreting the results of this analysis.

While the previous work of Doyle and colleagues [2] demonstrated consistent positive associations between patient-reported experience and clinical effectiveness, our study demonstrates that these associations are also true of users of mental health services. The idea that patient-reported experience of care could be a valuable indicator of quality of care in mental health services has been previously tested only in small studies with patients from a single service provider [7]. Our study builds up on this work and counters previously reported concerns about the ability of people with mental disorder to provide valid feedback about the quality of care they receive [10].

The main strength of this study is that we were able to examine the relationship between patient experience and quality of care across a large number of providers of mental health services throughout England and Wales. However, the study also has a number of limitations which need to be considered when interpreting the results. Firstly, the audit used two different samples for collecting case note data and for the patient survey, which did not allow us to determine whether individual patient ratings related to the quality of care provided to them personally by their Trust. The use of aggregate data from different samples of patients within each Trust may have underestimated the strength of the associations we might have seen, had both sets of data come from the same patient sample.

A second limitation of the study was that the measures of patient-rate experience and the global trust rating we used have not been validated. The global rating of trust quality of care was developed from standards for the treatment and management of people with schizophrenia published by the National Institute for Health and Clinical Excellence following a systematic review of published literature and extensive consultation with patients and providers [8]. However, the construct validity of the patient-rate experience questions and the global trust rating we used have not been tested. A further limitation of the patient-rate experience questions was that they were based on a single item.

While we found a moderate degree of association between patient satisfaction and patient-reported outcome in our analysis, only the latter was correlated with the independent rating of global quality of care provided Trusts. This suggests that patient-rated outcomes might be a better indicator of the quality of care that a service provides than patient-rated satisfaction with care. We also found that the two measures of patient-rated experience were associated with different elements of care delivered by participating Trusts. The more the services promoted the patients’ involvement in decisions about care and higher levels of compliance with recommended prescribing practice were associated with patients being more likely to state that services were improving their mental health. On the other hand, services that had higher compliance with recommendations that patients should have a care plan that was jointly developed to meet their needs had higher ratings on patient satisfaction with care.

These results support previous research in other healthcare settings which indicate that patients rate their experience and outcomes of care according to different criteria and highlight the importance of both in assessing the overall quality of care that healthcare services provide [11, 12].

In recent years concerns have been expressed about the burden that providers of healthcare services face in assuring the quality of care they deliver [13, 14]. The results of this study, together with other reports linking patient experience with service quality and safety [15, 16] suggest that greater use of patient surveys could be made in assuring the quality of healthcare and reducing the burden of data collection on service providers and assisting efforts to improve the quality of care that patients receive [17].

## Conclusion

In summary our findings highlight that, feedback from patients with severe mental disorders can provide important information about the quality of care they receive. Therefore, greater use of patient-reported experience measures should be made when assessing the quality of care provided to people with psychosis. Future work should focus on exploring the best way to combine patient views with audit data to obtain a comprehensive picture of the quality of care that services provide.

## Appendix

**Table 3:** List of the second round NAS standards

	Second round NAS standards
S1	Service users report that their experience of care over the past 12 months has been positive.
S2	Service users report positive outcomes from the care they have received over the past 12 months.
S3	Carers report satisfaction with the support and information they have been provided with to assist them in their role as a carer over the past 12 months.
S4	The following physical health indicators have been monitored within the past 12 months: i. body mass index, or waist circumference; ii. blood pressure; iii. Use of tobacco; iv. use of alcohol; v. substance misuse; vi. blood levels of glucose and lipids (total cholesterol and HDL cholesterol); vii. History of cardiovascular disease, diabetes, hypertension or dyslipidaemia in members of the service user’s family.
S5	When monitoring within the past 12 months has indicated a need for intervention, the following have been offered to the service user or the treating clinician has made a referral for the service user to receive: i. advice about diet and exercise, aimed at helping the person to maintain a healthy weight; ii. treatment for hypertension; iii. Treatment for diabetes; iv. treatment for dyslipidaemia; v. help with smoking cessation; vi. help with reducing alcohol consumption; vii. Help with reducing substance misuse.
S6	The service user has been provided with evidence-based, written information (or an appropriate alternative), in an accessible format, about the antipsychotic drug that they are currently prescribed.
S7	The service user was involved in deciding which antipsychotic was to be prescribed, after discussion of the benefits and potential side-effects.
S8	The service user is currently only prescribed a single antipsychotic drug (unless they are in a short period^a^ of overlap while changing medication or because clozapine is co-prescribed with a second antipsychotic) and a rationale for this has been documented.
S9	The current total daily dose of antipsychotic drug does not exceed the upper limit of the dose range recommended by the BNF. If it does, the rationale for this has been documented.
S10	If there was no or inadequate response to the first antipsychotic drug prescribed after a minimum of 4wks at optimum dose^b^: i. medication adherence was investigated and documented; ii. the potential impact of alcohol or substance misuse on response was investigated and documented.
S11	If there was no or inadequate response to the first antipsychotic drug within 8 weeks, part of which was at optimum dose^b^ the first antipsychotic drug was stopped and a second antipsychotic drug given.
S12	If there was no or inadequate response to two antipsychotic drugs, one of which should be a second-generation antipsychotic at optimum dose^b^, clozapine was offered.
S13	If there was no or inadequate response to treatment despite an adequate trial of clozapine^c^, a second antipsychotic was given in addition to clozapine for a trial period of at least 8 weeks at optimum dose^b^.
S14	a. CBT has been offered to all service users. b. Family intervention has been offered to all service users who are in close contact with their families.
S15	Each service user has a current care plan
S16	Each service user knows how to contact services if in crisis

^a^ Short period: Up to 6 weeks

^b^ Optimum dose: Up to three-quarters of BNF maximum or until side effects preclude further dose increase

^c^ Adequate trial of clozapine: At least 8 weeks at optimum dose with clozapine plasma concentration checked
